# A compelling demonstration of why traditional statistical regression models cannot be used to identify risk factors from case data on infectious diseases: a simulation study

**DOI:** 10.1186/s12874-022-01565-1

**Published:** 2022-05-20

**Authors:** Solveig Engebretsen, Gunnar Rø, Birgitte Freiesleben de Blasio

**Affiliations:** 1 Norwegian Computing Center, Oslo, Norway; 2 Department of Method Development and Analytics, Norwegian Institute of Public Health, Oslo, Norway; 3 Oslo Centre for Biostatistics and Epidemiology, Department of Biostatistics, Institute of Basic Medical Sciences, University of Oslo, Oslo, Norway

**Keywords:** Relative risk, Communicable diseases, Infectious diseases, Regression models, Overrepresentation

## Abstract

**Background:**

Regression models are often used to explain the relative risk of infectious diseases among groups. For example, overrepresentation of immigrants among COVID-19 cases has been found in multiple countries. Several studies apply regression models to investigate whether different risk factors can explain this overrepresentation among immigrants without considering dependence between the cases.

**Methods:**

We study the appropriateness of traditional statistical regression methods for identifying risk factors for infectious diseases, by a simulation study. We model infectious disease spread by a simple, population-structured version of an SIR (susceptible-infected-recovered)-model, which is one of the most famous and well-established models for infectious disease spread. The population is thus divided into different sub-groups. We vary the contact structure between the sub-groups of the population. We analyse the relation between individual-level risk of infection and group-level relative risk. We analyse whether Poisson regression estimators can capture the true, underlying parameters of transmission. We assess both the quantitative and qualitative accuracy of the estimated regression coefficients.

**Results:**

We illustrate that there is no clear relationship between differences in individual characteristics and group-level overrepresentation —small differences on the individual level can result in arbitrarily high overrepresentation. We demonstrate that individual risk of infection cannot be properly defined without simultaneous specification of the infection level of the population. We argue that the estimated regression coefficients are not interpretable and show that it is not possible to adjust for other variables by standard regression methods. Finally, we illustrate that regression models can result in the significance of variables unrelated to infection risk in the constructed simulation example (e.g. ethnicity), particularly when a large proportion of contacts is within the same group.

**Conclusions:**

Traditional regression models which are valid for modelling risk between groups for non-communicable diseases are not valid for infectious diseases. By applying such methods to identify risk factors of infectious diseases, one risks ending up with wrong conclusions. Output from such analyses should therefore be treated with great caution.

**Supplementary Information:**

The online version contains supplementary material available at 10.1186/s12874-022-01565-1.

## Background

Identifying overrepresented groups in infectious disease case statistics is important to guide targeted interventions. Previous studies have shown that interventions are most effective if they are targeted towards high-risk groups [1, 2]. If the elevated risk of infection can be attributed to intervenable causes, then targeted interventions can be implemented to eliminate these causes as part of the mitigation process.

During the COVID-19 outbreak, many studies have investigated potential risk factors for infection by using traditional statistical methods on data of the occurrence of infection in different groups [3–11]. As a motivating example in this article, we will consider studies investigating overrepresentation of foreign-born, immigrants, and certain ethnic minorities among individuals infected with COVID-19 [3–10]. Some suggested explanations include that these individuals are disproportionately overrepresented in specific groups of the population with higher risk of infection; they typically live in more crowded households, have lower socioeconomic status, have less access to health care and insurance, are at elevated risk for other underlying diseases, and are overrepresented in occupations with high exposure [3, 4, 12, 13].

To understand whether overrepresentation in such individual risk factors can explain the overrepresentation in cases, different studies have applied statistical regression models [3–9]. Common for these studies is that they find an effect of ethnicity or country of birth, even after adjusting for confounding/mediating factors associated with an elevated risk of infection, like socioeconomic status and household size. In these studies, the research question is often framed in terms of the direct effect of ethnicity on infection, therefore they want to control for known mediators and confounders. In our example, we thus also consider a mediating variable, but the numerical results would be identical in a situation where one would control for a confounding variable instead.

Traditional statistical regression methods can be applied to identify risk factors associated with different medical conditions, which in turn can provide insights into identifying potential causes for the condition. These methods are, as we will show, not in general suitable for infectious diseases. Infectious diseases differ from non-communicable diseases as there is no simple relationship between an increased risk of infection and the number of individuals infected.

Another related problem is that data from infectious diseases violate the crucial assumption for regression models of independent observations. This dependence is not easy to adjust for through, for example, cluster or time series analyses. Because transmissible diseases are acquired through contacts, the contact pattern and social network are the most important explanatory variables for infectious diseases [14]. Hence it is not only your individual risk factors that are important for your risk of infection, but also the properties/risk factors of your social network. This study will separate between individual risk factors that only include covariates related to the individual, and properties related to the social network. We will refer to the first as individual risk factors, and the latter as properties/risk factors of the individual’s contacts. As the disease outcome on an individual directly depends on the outcomes (and thus exposures) of other individuals in the population, we can both have direct effects on the individual due to their individual risk factors, and indirect effects of different exposure variables through the population due to the risk factors of the individual’s contacts [15–17]. In this study, we focus on estimating direct effects of exposures.

In this study, we will employ state-of-the art statistical methods to analyse data on communicable diseases by standard regression methods. By standard/traditional regression methods, we refer to readily available methods in statistical software which do not account for the detailed contact structure nor the dynamics of disease transmission. Though we refer to the methods as regression methods, other statistical techniques like statistical tests and ANOVA analyses are subject to the same problems if the contact structure and transmission dynamics are not considered.

One of the key reasons for using regression models is to adjust relationships for confounders or mediators, allowing estimates of direct effects of an exposure. In studies addressing the causes of why ethnic minorities are disproportionately affected by coronavirus disease, this is a main aim. In regression models, one can estimate the effect of a change in a variable on another variable, adjusted for other variables, in terms of the regression coefficient. Hence, by interpreting the regression coefficient, one can answer questions like: what is the risk of becoming sick in group A compared to group B if the factors C, which are differentially represented in the groups, would have been equally distributed. For infectious diseases, adjusting for potential confounders can be even more problematic than investigating univariant relationships because kinships, households, social and cultural structures shape human-to-human contact patterns, and hence the infection dynamics.

Assortative mixing, meaning a preference for individuals to have contacts with others that share characteristics or origins, is common in social networks. For example, this has been shown for traits like gender, age, occupation, religion, obesity, smoking, number of contacts, happiness, and negative vaccine sentiments [18–23]. Importantly, preferential mixing by ethnicity and immigrant status is well documented [18, 24, 25]. Hence, since certain immigrants/ethnic minorities belong to a high-risk group for infection, and typically have strong social ties within their group, individuals from these groups can be at higher risk of infection, even if they exhibit low-risk characteristics at the individual level.

In this study, we have constructed a simulation experiment to investigate the consequences of applying traditional statistical regression methods to analyse individual risk of infectious diseases. We use a simple, population-structured SIR-model [1] (susceptible-infectious-recovered), a general and well-established model for the spread of respiratory infections with acquired immunity, assuming random mixing within the population groups. We show that both point estimates and significance tests from regression models are likely to be wrong when applied to infectious diseases, and that it is necessary to take the data generation process into account. Although this study is motivated by the studies on risk factors for COVID-19 among immigrants, our results and conclusions are more general and relevant in other settings, specifically when the contact pattern is assortative.

## Methods

### Framework

We consider analyses where the goal is to use observed counts or prevalence of infection in various groups to learn about the underlying parameters of disease transmission. In this paper we analyse a simplified SIR-model where we divide the population into first two and then extend to four sub-groups. The two-group setting is the most parsimonious setting considered and is used to illustrate the relationship between individual and group-level risks, whether one can use a fitted regression model for contrafactual predictions to generalise to other group sizes, and the dependence between the incidence ratio in the two groups. The four-group setting is motivated by the studies of ethnicity and COVID-19 and is used to study the estimated regression coefficients from observational data.

We assume that we have data on the number of infected and total population sizes for each sub-group (individual-level data will give similar results) at timepoint t. In this simplified setup, either four or sixteen key parameters control the transmission dynamics between the two or four sub-groups, respectively. These parameters, ${\beta }_{ij}$, typically derived from contact studies, are summarised in a Who-Acquires-Infection-from-Whom matrix as usual in infectious disease modelling, where ${\beta }_{ij}$ is the rate of transmission from an individual in group $j$ to an individual in group $i$. We assume here that all groups have the same duration of infectiousness.

An important distinction is that “high-risk” can in this setup be due to at least three different factors that alone or in combination can explain an overrepresentation of cases in one of the sub-groups. We can decompose each ${\beta }_{ij}$ as ${\beta }_{ij}={inf}_{j}\times {c}_{ij}\times {susc}_{i}$, where ${inf}_{j}$ is the infectivity of group $j$, ${c}_{ij}$ is the number of contacts group $i$ has with group $j$ per time unit, and $sus{c}_{i}$ is the susceptibility of group $i$. Hence, we in general expect higher incidence in a group with higher susceptibility, or if the group has overall more contacts. We also expect higher incidence in a group with higher infectivity, if there are more contacts within than between groups. These three different potential causes of increased infection prevalence may have different effects on the overall disease dynamics in the population. In this study, we assume for simplicity that ${inf}_{j}=1$ in all simulations.

We define ${c}_{ij}$ as the total contact rate between groups $i$ and $j$, hence depending on the population sizes in the groups. It is easier to specify our scenarios in terms of ${p}_{ij}$, which is the relative frequency of contacts between individuals in groups $i$ and $j$. We specify a situation in which individuals have twice as many contacts within their group as between groups by $${p}_{ij}=\left(\begin{array}{cc}2/3& 1/3\\ 1/3& 2/3\end{array}\right).$$

This matrix is related to ${c}_{ij}$ as follows:

$${c}_{ij}=\frac{{p}_{ij}}{{w}_{i}},{w}_{i}=\frac{{\sum }_{j}{p}_{ij}{N}_{j}}{N{C}_{i}},$$

where ${C}_{i}$ is the total relative contact rate for group $i$. If all the groups have the same number of contacts, we have ${C}_{i}=1$. Hence, the ${c}_{ij}$ are calculated from the ${p}_{ij}$ in a way that ensures that the total number of contacts per time unit for everyone in group $i$ is given by ${C}_{i}$. In all settings except the last one (defined as case 4), we use ${C}_{i}=1$, such that all groups have the same total contact rate. Hence, when ${C}_{i}=1$, the ${c}_{ij}$ are calculated from the ${p}_{ij}$, ensuring that all groups have the same total number of contacts in the population per time unit.

In all simulations, we start with 0.1% infected in each group as our initial conditions.

### Simulated population

#### Two sub-groups

We simulate data in a population with $N=100 000$ citizens. We first split the population into two sub-groups $\mathrm{A}$ and $\mathrm{B}$, and we assume a higher susceptibility in group $\mathrm{B}$ than in group $\mathrm{A}$, such that $sus{c}_{B}=a\cdot {susc}_{A}$, where $a\ge 1$. We let ${N}_{A}=90 000$ and ${N}_{B}=10 000$ be the number of individuals in groups $\mathrm{A}$ and $\mathrm{B}$, respectively. The relative contact matrix is defined by

$$\left(\begin{array}{cc}{p}_{AA}& {p}_{AB}\\ {p}_{BA}& {p}_{BB}\end{array}\right),$$ where ${p}_{ij}$, $i,j\in A,B$ is the relative number of contacts group $i$ has with individuals in group $j$.

As an example of a contact structure in this population, let ${C}_{A}={C}_{B}=1, {p}_{AB}={p}_{BA}=1/3,{ p}_{AA}={p}_{BB}=2/3$. This corresponds to a setting where all individuals have the same total number of contacts, but twice as many contacts within their own sub-group. Plugging in the quantities we get ${{c}_{AA}=1.05, c}_{AB}=0.53, {c}_{BA}=0.91,{c}_{BB}=1.82.$

##### ***Cases***

We simulate two cases in the two-group setting, case 1 and case 2.

**Case 1**: We let $a=1.2$. We vary the basic reproduction number ${R}_{0}$ (or, equivalently, ${susc}_{A}$) and the timepoint for which we compare the outcome (T). We assume no contact between the groups, so $$\left(\begin{array}{cc}{p}_{AA}& {p}_{AB}\\ {p}_{BA}& {p}_{BB}\end{array}\right)=\left(\begin{array}{cc}1& 0\\ 0& 1\end{array}\right)$$. We compute the incidence rate ratio (hereafter denoted as relative risk) resulting from the simulations, that is, the proportion infected in sub-group $B$ divided by the proportion infected in sub-group $A$. For non-communicable diseases, one would expect a one-to-one relationship between individual risk of disease and relative risk, i.e. a relative risk of 1.2.

**Case 2**: We set ${susc}_{A}$ such that for $a=1$, ${R}_{0}=0.9$, and then vary $a$. See supplementary material for the expression for ${R}_{0}$. We assume random mixing between the groups, so $$\left(\begin{array}{cc}{p}_{AA}& {p}_{AB}\\ {p}_{BA}& {p}_{BB}\end{array}\right)=\left(\begin{array}{cc}1/2& 1/2\\ 1/2& 1/2\end{array}\right)$$. We study the results for time point T = 200 days, which for most parameter choices in the paper corresponds to after the epidemic has burnt out. We compare two different outcomes:

A) We investigate whether the predictions of a Poisson regression model (see Sect. Poisson regression model) fitted to simulated data on the number infected in each group from a setting with a difference between the two groups can be used to predict the total number of infections in the setting with no difference between the groups. Specifically, the fitted regression model is used to predict the proportion infected in the contrafactual scenario where the entire population belongs to the low-risk group $A$, that is, ${N}_{A}=100 000, {N}_{B}=0$. We compare this prediction with the simulations when the whole population is in $A$. For non-communicable diseases, one would expect no discrepancy between the simulations and the predictions from the fitted model.
B) We plot the proportion infected in the low-risk group when we vary the susceptibility in the high-risk group. If individual risks of infection only depended on individual characteristics, one would expect the proportion infected in the low-risk group to be independent of the properties of the high-risk group.

#### Four sub-groups

We split the population into four groups, ${A}_{h}$, ${A}_{l}$, ${B}_{h}$, and ${B}_{l}$, inspired by the recent analyses of ethnicity and risk of COVID-19 infection. We assume two ethnicity groups $A$ and $B$, and that one ethnicity group ($B$) is disproportionately represented in a high-risk group. Hence, the two ethnicity groups are divided into two risk groups, with one risk group ($h$) having a higher risk than the other ($l$). This could for example represent a high-risk occupation. As before, we let ${N}_{A}=90 000$ individuals, and ${N}_{B}=10 000$ individuals. We further assume that 10% and 50% of the individuals in ethnicity groups $A$ and $B$ are in the high-risk group, respectively. Hence, we let group ${A}_{h}$ be the ${N}_{{A}_{h}}=9 000$ individuals in ethnicity group $A$ with the high individual risk, group ${A}_{l}$ be the ${N}_{{A}_{l}}=81 000$ individuals in ethnicity group $A$ with low individual risk, group ${B}_{h}$ be the ${N}_{{B}_{h}}=5 000$ individuals in ethnicity group $B$ with high individual risk, and finally ${B}_{l}$ be the ${N}_{{B}_{l}}=5 000$ individuals in ethnicity group $B$ with low individual risk.

The contact matrix is defined by the contacts between the risk levels $h$ and $l$, and the contacts between the ethnicity groups $A$ and $B$. Hence, let

$$\left(\begin{array}{cc}{p}_{hh}& {p}_{hl}\\ {p}_{lh}& {p}_{ll}\end{array}\right)$$ be the relative contact matrix between the risk levels, where ${p}_{ij}$, $i,j\in h,l$ is the relative number of contacts risk group $i$ has with individuals in risk group $j$. Further, let

$$\left(\begin{array}{cc}{p}_{AA}& {p}_{AB}\\ {p}_{BA}& {p}_{BB}\end{array}\right)$$ be the relative contact matrix between ethnicity groups $A$ and $B$, where ${p}_{ij}$, $i,j\in A,B$ is the relative number of contacts ethnicity group $i$ has with individuals in ethnicity group $j$. The relative contact matrix between the groups ${A}_{h}$, ${A}_{l}$, ${B}_{h}$, and ${B}_{l}$ is then defined by the outer product of these two matrices, such that

$$\left(\begin{array}{cc}\begin{array}{cc}{p}_{{A}_{h}{A}_{h}}& {p}_{{A}_{h}{A}_{l}}\\ {p}_{{A}_{l}{A}_{h}}& {p}_{{A}_{l}{A}_{l}}\end{array}& \begin{array}{cc}{p}_{{A}_{h}{B}_{h}}& {p}_{{{A}_{h}B}_{l}}\\ {p}_{{A}_{l}{B}_{h}}& {p}_{{A}_{l}{B}_{l}}\end{array}\\ \begin{array}{cc}{p}_{{B}_{h}{A}_{h}}& {p}_{{B}_{h}{A}_{l}}\\ {p}_{{B}_{l}{A}_{h}}& {p}_{{B}_{l}{A}_{l}}\end{array}& \begin{array}{cc}{p}_{{B}_{h}{B}_{h}}& {p}_{{B}_{h}{B}_{l}}\\ {p}_{{B}_{l}{B}_{h}}& {p}_{{B}_{l}{B}_{l}}\end{array}\end{array}\right)=\left(\begin{array}{cc}\begin{array}{cc}{p}_{hh}{p}_{AA}& {p}_{hl}{p}_{AA}\\ {p}_{lh}{p}_{AA}& {p}_{ll}{p}_{AA}\end{array}& \begin{array}{cc}{p}_{hh}{p}_{AB}& {p}_{hl}{p}_{AB}\\ {p}_{lh}{p}_{AB}& {p}_{ll}{p}_{AB}\end{array}\\ \begin{array}{cc}{p}_{hh}{p}_{BA}& {p}_{hl}{p}_{BA}\\ {p}_{lh}{p}_{BA}& {p}_{ll}{p}_{BA}\end{array}& \begin{array}{cc}{p}_{hh}{p}_{BB}& {p}_{hl}{p}_{BB}\\ {p}_{lh}{p}_{BB}& {p}_{ll}{p}_{BB}\end{array}\end{array}\right).$$

We vary the amount of mixing within and between the ethnicity groups (assortativity). We define random mixing in ethnicity groups as a contact structure where every individual has the same probability of being in contact with any other person irrespective of ethnicity groups, while we denote an assortative contact structure as homogeneous mixing. We simulate with different levels of assortative mixing, defined by the relative number of contacts within to between the ethnicity groups. An assortativity of 1 then corresponds to random mixing with ${p}_{AA}={p}_{AB}={p}_{BA}={p}_{BB}=1/2$. An assortativity of 2 is defined as twice as many contacts within ethnicity groups, that is, ${p}_{AA}={p}_{BB}=2/3, {p}_{AB}={p}_{BA}=1/3$, where we divide by 3 for normalisation. More generally, an assortativity of $x$ is defined by $x$ times as many contacts within as between ethnicity groups, such that ${p}_{AA}={p}_{BB}=x/(x+1), {p}_{AB}={p}_{BA}=1/(x+1)$.

##### ***Cases***

We simulate two different cases in the four-group setting, cases 3 and 4, with varying definitions of the high-risk individuals. We set the parameters in both cases such that ${R}_{0}=1.3$. We study the results at T = 200 days.

**Case 3**: In case 3, the high-risk individuals are defined by a higher susceptibility than the low-risk individuals, in such manner that ${susc}_{{A}_{h}}={susc}_{{B}_{h}}=a\cdot sus{c}_{{A}_{l}}={a\cdot susc}_{{B}_{l}}$, with $a\ge 1$. We assume ${p}_{ij}=1/2$ for all $i,j\in h,l$ and vary $a$. This definition of elevated risk could correspond to closer contact, genetic or biological differences, fewer hygienic precautions, or a mixture of those effects. We compute how much of the relative risk of ethnicity group $B$ compared to $A$ can be explained by a higher proportion of high-risk individuals (see Sect. Unexplained relative risk). For non-communicable diseases, we would expect no unexplained relative risk. For $a=2$ we fit a Poisson regression model on the outcome of the simulations, adjusting for risk level and ethnicity. We investigate how the confidence interval (CI) of the regression coefficient related to ethnicity varies with assortativity.

**Case 4**: In case 4, we assume ${susc}_{A_h}={susc}_{A_l}={susc}_{B_h}={susc}_{B_l}=1$, but we let the high-risk individuals have more contacts than the low-risk individuals. The additional contacts are with other high-risk individuals, such that ${p}_{hh}=d\cdot {p}_{hl}={d\cdot p}_{lh}=d\cdot {p}_{ll}$, where $d\ge 1$. We assume ${p}_{hl}={p}_{lh}={p}_{ll}=1$ and let $d=3$. To account for the increased number of contacts, we now use ${C}_{{A}_{h}}={C}_{{B}_{h}}=1, {C}_{{A}_{l}}={C}_{{B}_{l}}=1.28$. As for case 3, we fit a Poisson regression model to the simulation outcome and investigate the CI for the ethnicity regression coefficient when varying assortativity. This definition of elevated risk could be due to larger households, less adherence to social distancing advice, or an occupation that requires more contacts.

### Transmission model

We simulate disease spread by a stochastic SIR-model [1]. Let ${S}_{i}$, ${I}_{i},$ and ${R}_{i}$ be the number of susceptible ($S$), infectious ($I$), and recovered ($R$) individuals in group $i$. The following set of difference equations describes the disease development over time ${S}_{i}\left(t+\Delta t\right)= {S}_{i}\left(t\right)-{X}_{1},$ ${I}_{i}\left(t+\Delta t\right)= {I}_{i}\left(t\right)+{X}_{1}-{X}_{2},$ where ${X}_{1}\sim \mathrm{Binom}\left({S}_{i}\left(t\right),\Delta t\sum_{j}\frac{{susc}_{i}{p}_{ij} in{f}_{j}{I}_{j}}{N}\right),$ and ${X}_{2}\sim \mathrm{Binom}\left({I}_{i}\left(t\right), {\Delta }_{t}\gamma \right),$ where $1/\gamma =3$ days is the assumed duration of the infectious period, $\Delta t=0.2$ is the time-step used in the simulations, and $i=A, B$ in the two-group setting, and $i={A}_{h}$, ${A}_{l}$, ${B}_{h}$, ${B}_{l}$ in the four-group setting. We assume a constant population size so that ${R}_{i}={N}_{i}-{S}_{i}-{I}_{i}$.

### Analysis of simulation results

#### Poisson regression model

We fit a Poisson regression model on the outcome of the disease simulation, here exemplified in the four-group setting. We include ethnicity and risk level as covariates, resulting in the following regression model ${\mathrm{log}{\mu }_{i}=\mathrm{log}{N}_{i}+{\beta }_{0}+\beta }_{e}{e}_{i}+{\beta }_{r}{r}_{i}$, where ${Y}_{i}\sim \mathrm{Poisson}\left({\mu }_{i}\right)$ denotes the number of infected individuals in group $i$, ${e}_{i}$ and ${r}_{i}$ denote ethnicity and risk group status for group $i$, respectively, and as before, ${N}_{i}$ are the population sizes of each group used as an offset. We are interested in the estimated regression coefficient ${\beta }_{e}$, which quantifies the effect of ethnicity. We let ethnicity group $B$ and $l$ be the reference levels for ethnicity and risk level, respectively. We assume a standard significance level of 0.05.

#### Unexplained relative risk

Since a higher proportion of ethnicity group $B$ belongs to the high-risk group, we expect a larger infected proportion in ethnicity group $B$. We denote the explained relative risk in ethnicity group $B$ compared to $A$ as the relative risk which can be explained by a higher proportion in the high individual risk group. This can be computed from the proportion of high-risk individuals in each ethnic group, together with the observed relative risk between the high and low-risk group. Specifically, let ${R}_{{A}_{h}}, {R}_{{A}_{l}, }, {R}_{{B}_{h}},$ and ${R}_{{B}_{l}}$ be the total proportion of infected individuals in each group. The explained relative risk, $ER$, is then given as $ER=(0.5+0.5{RR}^{r})/(0.9+0.1{RR}^{r})$, where ${RR}^{r}=({R}_{{A}_{h}}+{R}_{{B}_{h}})/({R}_{{A}_{l}}+{R}_{{B}_{l}})$, is the observed relative risk between the high- and low-risk groups. The unexplained relative risk, $UR$, in ethnic group $B$ is then given by the observed relative risk in ethnic group $B$, minus the explained relative risk, that is $UR=({R}_{{B}_{h}}+{R}_{{B}_{l}})/({R}_{{A}_{h}}+{R}_{{A}_{l}})-ER$. For non-communicable diseases, we would expect no unexplained relative risk.

#### Estimating from the data-generation model using approximate Bayesian computation

In addition to analysing the simulated data with regression models, we apply a simple Markov Chain Monte Carlo approximate Bayesian computation (ABC-MCMC) algorithm [26] to estimate the risk parameters in the groups from the simulations. We use these simulations to explore if we can obtain the correct parameters when the true data-generating model is accounted for. Details are provided in the supplementary material.

## Results

### Case 1

**Fig. 1:**
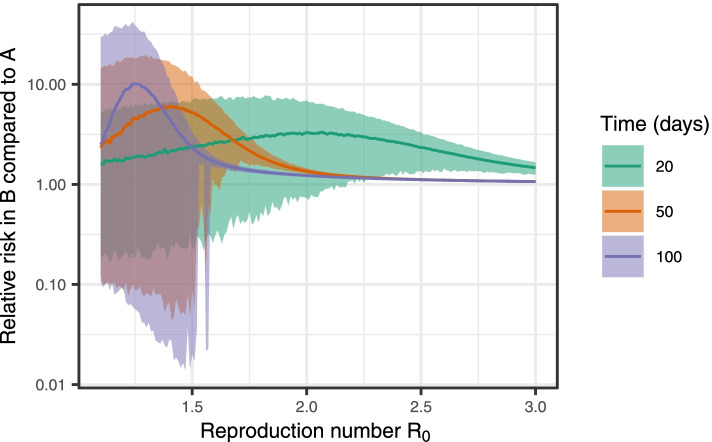
Case 1. Proportion infected in group $\mathrm{B}$ divided by the proportion infected in group $\mathrm{A}$ when varying $R_{0}$. Median and 95% CI based on 1000 simulations

Figure 1 shows the relative risk obtained in the simulations when ${R}_{0}$ is varied and the individual risk in group $B$ is 20% higher than the individual risk in group $A$ for different simulation times T. We note that there is no simple relationship between the difference in individual risk and the overrepresentation at group level in the simulations; when varying ${R}_{0}$, the overrepresentation varies between 0 and 10. There is a large spread of relative risk values for each value of ${R}_{0}$ due to the stochastic nature of the transmission model. As ${R}_{0}$ becomes large, the overrepresentation becomes small, as most of the population is infected. For higher ${R}_{0}$, the overrepresentation is in general larger earlier in the outbreak (low T) than later. For lower ${R}_{0}$, the overrepresentation is in general larger later in the outbreak (high T) than early in the outbreak. The disease dynamics for this set of models are provided in the supplementary material, section Infection dynamics in cases 1 and 2.

### Case 2

**Fig. 2:**
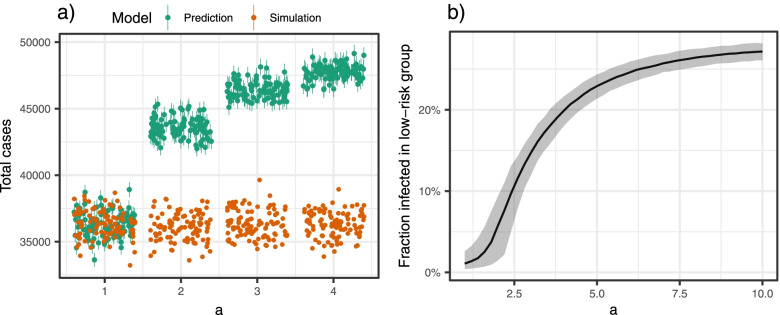
Case 2. **a**) Predictions from the fitted Poisson regression model in green and disease simulations in orange for a population with only low-risk individuals. **b**) Fraction infected in the low-risk group when keeping all parameters fixed except the susceptibility in the high-risk group ($\mathrm{a}$). Median and 95% CI based on 1000 simulations

Figure 2a shows the discrepancy between the predictions from the regression model and the model simulations when we use the regression model fitted on data based on two groups with different susceptibilities to predict the total number of cases if we only had one group (low risk), when the difference in susceptibility is varied. We note that the regression model’s predictions significantly overestimate the proportion of infected when everyone belongs to the low-risk group. The problem increases when the relative susceptibility between the groups ($a$) increases. When there is no difference between the high- and low-risk group, the point predictions from the regression model perform well in this simple two-group setting. However, the figure clearly shows that the prediction intervals from individual simulations do not adequately cover the spread in simulations, indicating that the Poisson regression underestimates the uncertainty. For non-communicable diseases and other settings where Poisson regression is applicable, we would expect no discrepancy.

Figure 2b shows how the proportion of infected in the low-risk group depends on the properties of the high-risk group. The larger the susceptibility in the high-risk group, the larger proportion infected in the low-risk group. We note that we need a large enough $a$ to sustain an epidemic in the high-risk group. We also note a saturation effect in $a$, such that above a certain level, the fraction infected in the low-risk group is almost constant when $a$ increases. The ratio of infected in the low-risk group increased from approximately 0 to almost 0.3, through increasing the susceptibility of the high-risk group. The disease dynamics for case 2 are provided in the supplementary material, section Infection dynamics in cases 1 and 2.

Note that we have chosen to illustrate the results for T = 200, but for other time points, the results would likely be different, as illustrated in Fig. 1.

### Cases 3 and 4

**Fig. 3:**
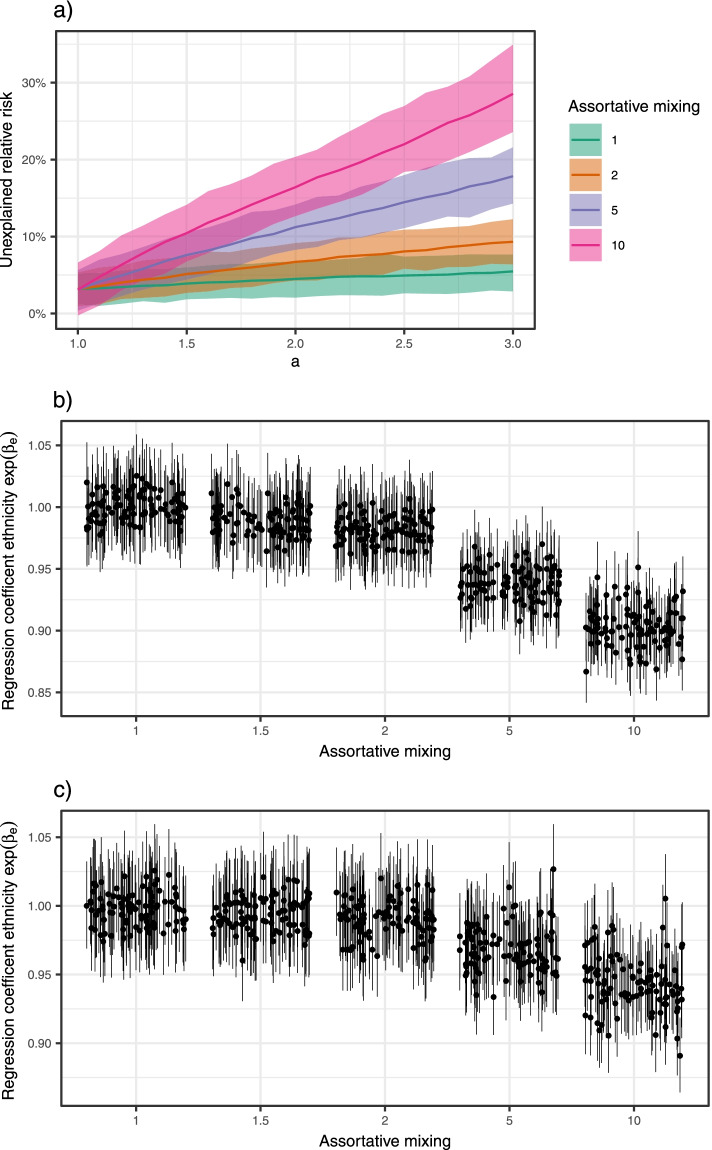
Cases 3 and 4. **a**) Unexplained relative risk as a function of the relative susceptibility of the high- to low-risk individuals ($\mathrm{a}$). The different lines correspond to different levels of assortative mixing. Median and 95% CIs based on 500 simulations. **b**) 95% CIs for the effect of ethnicity $\mathrm{exp}(\beta_{e})$ from the fitted Poisson regression model for varying levels of assortative mixing. The high-risk individuals are defined by a larger susceptibility than the low-risk individuals (case 3). There are 100 simulations for each assortativity level. **c**) Same as **b**), except the high-risk individuals are defined by a larger total number of contacts (case 4)

Figure 3a shows how the unexplained relative risk for ethnicity group $B$ increased when we increased the relative risk between the high- and low-risk group. The effect is more prominent when the assortative mixing within ethnic groups is larger. For non-communicable diseases, we expect no unexplained relative risk.

Figures 3b and c show the CI for the ethnicity regression coefficient when the proportion of contacts within the same ethnicity groups increases for cases 3 and 4, respectively. Since we know the truth in the simulation, we know that ethnicity does not affect the individual risk of infection. For random mixing, the regression analysis resulted in the truth—no effect of ethnicity (CIs centred at 1). However, the higher the tendency for contacts within the ethnicity groups, the higher the estimated effect of ethnicity. With a large enough degree of assortative mixing, we find an ethnicity coefficient significantly different from 1. Note that we are focussing on the significance of the coefficients and not on the effect sizes. As illustrated for case 1 (cf. Figure 1), the effect sizes are highly dependent upon which time point is used to compare the relative risks, as these cannot be interpreted as estimates of individual-level properties. This is the case for both cases 3 and 4. The effect is larger when we increase the susceptibility in the high-risk group than when we increase the number of contacts, but the results for the two different definitions of the high-risk group are qualitatively very similar.

In the supplementary material we show that we can apply the ABC-MCMC algorithm together with the simulation model to accurately estimate the transmission parameters. To accurately estimate some of the parameters, the true transmission model and the other parameters are needed. For example, one needs to assume a value, or a range of possible values, for the assortativity.

### Validity of traditional methods

The results above show that, in general, one cannot use traditional statistical methods to estimate the individual-level effects from population-level infectious disease outcomes. While this is true, if we are interested in explaining factors that are associated with increased risk, such methods can be adequate and can give a good approximation when the dynamics and feedback characterising the spread of infectious diseases are not important.

To illustrate, we consider a deterministic version of the SIR model with two sub-groups, with the same population sizes in the groups, and a relative difference in susceptibility by a factor $a$ in group $B$ compared to group $A$. We further assume that we start with the same number infected in each group. In this example, the number of infectious individuals ${I}_{i}$ is given by the following differential equations:

$$\frac{d{I}_{i}}{dt}=\frac{{\lambda }_{i}S}{N}-\upgamma {I}_{i},{\uplambda }_{i}=\sum_{j}sus{c}_{i}{c}_{ij}{I}_{j}.$$

Under random mixing, we find ${\lambda }_{i}=sus{c}_{i}I$, where $I={I}_{A}+{I}_{B}$ is the total number of infected individuals in the population. During the early phase of an outbreak ($S\approx N$), we can approximate the number of new cases in $\Delta t$ by:

$$in{c}_{A}=sus{c}_{A}\times I\times\Delta t, in{c}_{B}=sus{c}_{A}\times a\times I\times\Delta t,$$

where $in{c}_{A}$ and $in{c}_{B}$ are the incidences in groups $A$ and $B$, respectively. We then find $RR=in{c}_{B}/in{c}_{A}=a.$ This approximation holds until ${S}_{i}/N$ becomes different in the two groups. The difference in susceptibility means that this ratio will change at different rates in the two groups. Therefore, after some time, the incidence ratio of observed cases will diverge from $a$.

One of the most important settings where traditional statistical methods are used to estimate relative risks of infection is in randomised controlled trials to estimate vaccine effect [27]. In this setting traditional methods will still work, since even if the mixing in the whole population might not be random, it has been shown that as long as the two groups we are comparing have the same contact structure, we are in a similar regime as described above [28, 29]. This requirement will be fulfilled by randomisation. As above, one might still get a biased estimate if a large fraction of the population is infected during the trial such that ${S}_{i}/N$ changes. Typically, these trials take part over a reasonably short time so the traditional methods will still likely be valid.

**Fig. 4:**
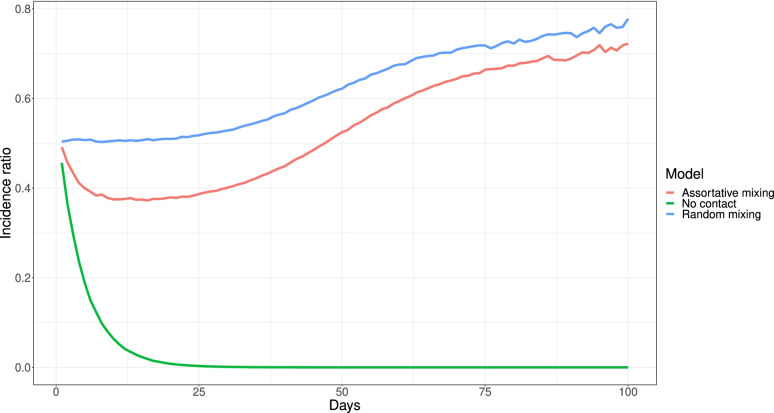
Incidence ratio comparison. Daily incidence ratio between two groups with a relative difference of susceptibility of 2. The $R_{0}=1.3$ in all settings

In the situation without random mixing, for example assuming twice as many contacts within as between groups, the main difference from the approximated relations above is that ${\uplambda }_{i}$ is no longer proportional to $I$. This means that although we could recover the individual effect from the population effect in the very beginning, the relation is broken immediately as soon as ${I}_{1}\ne {I}_{2}$, which will occur after the first generations of the disease spread. Figure 4 shows the ratio of the daily incidence in the two groups, simulated using the stochastic model with $a=0.5$. We consider three contact structures: assortativity of 2, random mixing, and no contact between groups (${p}_{AA}={p}_{BB}=1,{p}_{AB}={p}_{BA}=0$). With random mixing, the incidence ratio is approximately 0.5 for about 25 days, while for assortative mixing, the incidence ratio drops almost immediately to about 0.4. The drop is caused by the indirect protection in group $B$, since they have more contacts with individuals who are less likely to be infected. The setting with no contact between the groups shows an extreme effect of the indirect protection as for this model ${R}_{0}<1$ in group $B$, and hence there is no large outbreak in the group. For the randomly mixing case, or when comparing two groups with the same contact patterns, it is possible to analytically relate the population incidence ratio to the individual-level difference in susceptibility [28, 30].

## Discussion

Our simulations show that the standard traditional regression methods are not, in general, valid for infectious diseases. We show that the results from applying such methods to data on infectious diseases to identify risk factors are not interpretable. One can even risk ending up with qualitatively wrong results by using them. The main reason is that traditional regression methods do not consider the dependency between the cases. For the risk of acquiring directly transmissible infections, the indirect effects are the most important—unless you are exposed to the infection, your individual risk profile is irrelevant.

The interdependence between the individuals in transmission chains leads to a complex relationship between increases in transmissibility and the total number infected that depends on many parameters that either need to be measured or assumed.

We summarise the main take-home messages related to the use of traditional statistical methods as

1. There is no obvious relationship between increased individual-level risk of infection and the observed number of cases on population-level. This relation must be interpreted through untangling the transmission dynamics.
2. The regression coefficients are not interpretable. Estimates from regression analysis on observed relative risk will not estimate the underlying parameters of the transmission process.
3. There is no one-to-one relationship between individual risk and population risk. Estimates like regression coefficients cannot be interpreted in the standard way as how the incidence would change if we removed or changed a risk factor.
4. Individual risks are not identifiable as they depend on the properties of the rest of the population. The proportion of infected in the low-risk group increased significantly by only changing the susceptibility of the high-risk group.
5. As regression coefficients are not interpretable, this also means that adjusting for confounders and mediators is not possible in traditional ways. We cannot assess how much of the overrepresentation can be explained by other, correlated covariates.

In a simple setting of two sub-groups, one can conclude that if the relative observed risk between the two groups is different from one, then there is a difference between the two groups. However, it is not possible to use regression analysis assuming independence between the observations to assess the size of the effect and/or whether the difference is significantly different. In a more complex setting of more than two groups, we cannot conclude anything about the relative individual risks of infection between two groups, as there is potential confounding or mediation that we cannot adjust for, depending on the research question.

In our simulation experiment, we find a significant effect of ethnicity, even though the example was constructed such that the behaviour was identical in the two ethnicity groups. The only difference was the proportion of individuals belonging to a high-risk group, which we adjusted for in the regression analysis. The larger the tendency for mixing within ethnicity groups, the larger the estimated effect of ethnicity, while for random mixing, we correctly found no effect of ethnicity. It is thus clear that by applying a standard regression model, we have not properly adjusted for the mediation through the risk level variable and thus we have not been able to estimate the direct effect of ethnicity.

Multiple studies conclude an effect of ethnicity/country of birth/immigration status on the risk of infection of COVID-19, even after adjusting for socioeconomic variables and other potential risk factors [3–9]. As illustrated, regression models cannot be used to adjust for these variables, even if the variables were perfect measures of what one wishes to control for. We therefore conclude that great caution should be applied when interpreting such results.

Though we have chosen to illustrate our points by the example of the overrepresentation of COVID-19 among certain ethnic groups, our results will be valid in general when analysing infectious disease case data with traditional regression methods. Another important example arises in analysing vaccine efficacy from observational studies. Regression models have been used in observational studies to evaluate vaccine efficacy during COVID-19 [31–33]. As we have illustrated in this paper, it is generally not feasible to estimate the individual vaccine efficacy from aggregated observed counts of infection and compare infection risks. It is also not possible to adjust for confounding effects like age, a well-documented strong confounder with vaccination due to age-prioritised COVID-19 vaccination strategies. Age mixing is also known to be assortative, as seen for example from the POLYMOD study [34]. The effects of assortativity on bias in observational vaccination studies was also studied by simulation in [35]. However, randomised controlled trials on vaccine efficacy do not suffer from the same problem, as the independence assumption is more reasonable in a randomised controlled trial. Hence, there are settings when traditional techniques are applicable. However, if traditional techniques are to be used, they should be accompanied by an argument about why the situation under study falls into such a regime.

Recently, there have been developed methods for causal inference under different dependence structures which could be used to estimate effects of interventions like vaccination on infectious diseases [16, 17, 36, 37]. However, these methods typically require either specific experimental designs such that one can assume partial interference, that is, that the population can be divided into independent groups, or knowledge of the underlying social network.

In this study, we have only investigated the effect of violation of the independence assumption. A more general problem with many of these studies is the lack of a clear definition of risk factors, and an unclear specification of the research question. Whether a factor is found to be a risk factor or not for a specific condition depends on both how the research question and risk factor are defined [38]. For example, risk factors for becoming infected in the future may differ from risk factors of having been infected, as certain groups may have gained high immunity levels. To conclude about the (causal) meaning of the risk factor from an analysis, a proper understanding of the interplay between the different factors is necessary, through for example a directed acyclic graph of the problem. This is necessary to avoid so-called table 2-fallacies, where effects in multiple regression analysis can be misinterpreted, as discussed in [39]. Another general problem is measurement error and confounding [40], as many variables like socioeconomic status are hard to measure, and there is strong correlation between many covariates. The conclusions may also depend on the model specification through for example the response distribution and the assumed shape of the covariate effects. Moreover, in this study we have focussed on estimating the direct effect of an exposure on the disease outcome. One could also be interested in other parameters, like the total or indirect effect of an exposure [15–17]. This can for example be of key interest when analysing the effect of an intervention like vaccines, where one might be interested both in the direct protective effect of the vaccine on the individual, and on the indirect effect of protection through for example herd immunity. In an infectious disease modelling framework these indirect effects can be estimated from the individual level direct effects.

This study only considered how the overrepresentation might vary when the number of contacts and susceptibility differ between groups, assuming different underlying contact structures. Other factors which could affect overrepresentation are different importation rates, infectivity, and duration of infectious period. It is straightforward to extend our framework also to consider the effect of these three factors.

The population structure assumed in this study is overly simplified, and random mixing is a very strong assumption which does not reflect well a realistic contact structure. Similarly, we assume little heterogeneity between individuals within the same sub-group (e.g. the same duration of infectiousness, susceptibility, infectivity, and contact pattern). Our aim has thus not been to perform an exhaustive overview of parameters and how they may affect the results. The model is constructed primarily for a theoretical, academic purpose with focus on parsimony to illustrate a point. Our model is thus not meant to be used to conclude about causes for the observed overrepresentation of COVID-19 cases among ethnic minorities.

The fact that regression methods that do not consider transmission do not apply to infectious diseases is neither surprising nor novel. The bias of traditional measures like risk ratios and odds ratios was demonstrated in a study from 1991 [41] inspired by the AIDS epidemic, among several other studies [42–44]. However, recent statistical analyses of COVID-19 case data show the necessity of a reminder. Moreover, to our knowledge, our study is the first to consider this in a regression setting.

Different methods suitable for inference on infectious diseases have been proposed [45–48]. The problem with many of these methods is that they are hard to use and may require detailed data about the contact pattern of the population. Such data are rarely available, particularly for diseases which do not necessarily require direct physical contact but may spread through the environment through aerosols and droplets (e.g. COVID-19). In this paper, we show that if one can specify an assumed data-generating model, it could be possible to estimate some of the parameters of interest. Such methods require a lot of data, especially about contact structures, and must be tailored to each study context. For intervenable factors like for example vaccines, there have been suggested experimental designs which would allow the use of classical statistical methods to analyse the effects of the factors, as mentioned above. However, for immutable properties (like ethnicity), we believe that it is better to use pure descriptive analyses rather than perform regression methods where the coefficients are not interpretable. In particular, one should not draw conclusions or interpret the effects from such studies. Hence, there is a need for popularised, easy-to-use methods applicable to inference on infectious diseases, which can be applied to the data typically available at hand. There is also a need for continuous ongoing surveys to collect data on social contacts and behaviour. In recent years and particularly during COVID-19, new, alternative data streams like mobile phones have been used to inform models, enabling detailed real-time information on behaviour.

In this article we only consider using regression models to identify and measure risk-factors for infection. There are many other applications of regression models in infectious diseases that are not affected by the problems discussed here, including using regression to estimate the growth rate of cases and for anomaly detection in surveillance.

## Conclusions

We conclude that using standard methods like Poisson regression models to study overrepresentation of different groups does not make sense for infectious diseases. If the methods developed for non-communicable diseases are used to analyse infectious diseases, one can risk ending up with the wrong qualitative conclusions.

## Supplementary Information

**Additional file 1.**

## Data Availability

All the source code used in the study is publicly available at: https://github.com/Gulfa/pitfalls. All analyses were performed in R [49] using the odin.dust R-package [50]. Data sharing is not applicable to this article as no datasets were generated or analysed during the current study.

## Abbreviations

SIR: Susceptible-infectious-recovered
CI: Confidence interval
ABC-MCMC: Markov Chain Monte Carlo approximate Bayesian computation
