# Supplementary material for: A compelling demonstration of why traditional statistical regression models cannot be used to identify risk factors from case data on infectious diseases: a simulation study
Source: BMC Med Res Methodol. 2022 May 20;22:146. doi: 10.1186/s12874-022-01565-1 (PMC9123765; doi:10.1186/s12874-022-01565-1)
Supplement: Supplementary file 1 — Additional file 1. [file 12874_2022_1565_MOESM1_ESM.pdf]

Supplemental material for:

A compelling demonstration of why traditional statistical regression models cannot be used to identify risk factors from case data on infectious diseases: a simulation study

Solveig Engebretsen[1*†], Gunnar Rø[2†], Birgitte Freiesleben de Blasio[2,3]

[1]Norwegian Computing Center, Oslo, Norway

[2]Department of Method Development and Analytics. Norwegian Institute of Public Health, Oslo, Norway

[3]Oslo Centre for Biostatistics and Epidemiology, Department of Biostatistics, Institute of Basic Medical Sciences, University of Oslo, Norway

[*]Corresponding author. E-mail: solveig.engebretsen@nr.no.

† The authors contributed equally.

S1 Methods

S1.1 Expression for reproduction number

We provide the expression for the basic reproduction number, R_0, in the setting with four sub-groups, based on the largest eigenvalue of the next-generation matrix [1]. In this setting the next-generation matrix, R_{ij}, is defined by:

$$R_{ij} = sucs_i \times c_{ij} \times \frac{N_i}{N}.$$

We then choose a value of the overall susceptibility such that we can run with the desired basic reproduction number. The value is found numerically.

S1.2 Estimating from the data-generation-model using ABC

We investigate whether a simple rejection Markov Chain Monte Carlo approximate Bayesian computation algorithm (ABC-MCMC) [2] can estimate the true effect of ethnicity in the case 3 setting with four sub-groups and $a = 1.2$. We define the ethnicity effect by the parameter β_e such that $\beta_e \cdot susc_{A_h} = susc_{B_h} = a \cdot \beta_e \cdot susc_{A_l} = a \cdot susc_{B_l}$, that is, the high-risk group has an increased susceptibility by a factor $a = 1.2$ and ethnicity group B has an increased susceptibility by a factor β_e. The aim is to understand whether it is possible to obtain the correct parameters when the data-generating model is taken into account. The idea behind ABC is to obtain parameters which provide simulations that are close to the observed data. The observed data are taken from a simulation with the model. We will assume that all parameters except β_e and the ethnicity assortativity are known. We assume that the basic reproduction number is known, and hence calculate $susc_{A_l}$ from the reproduction number. Hence, this is an ideal situation for the parameter estimation, and the results are likely to be less accurate in a real data situation. We assume two parameter values for β_e, $\beta_e = 1.0$ and $\beta_e = 1.05$ and that the ethnicity assortativity is 10. We use a

basic reproduction number of 1.3 and the contact structure between risk groups $\begin{pmatrix} p_{hh} & p_{hl} \\ p_{lh} & p_{ll} \end{pmatrix} = \begin{pmatrix} 1/2 & 1/2 \\ 1/2 & 1/2 \end{pmatrix}$. We also assume the same total number of contacts in all four groups, so $C_{A_h} = C_{A_l} = C_{B_h} = C_{B_l} = 1$. We first estimate both β_e and the ethnicity assortativity, then we consider the case with a known assortativity and only estimate β_e. We assume independent priors. For β_e we assume a rather wide normal prior distribution with mean 1.0 and variance 9, truncated at 0. For the assortativity we assume a uniform prior from 0.05 to 20. We use the ABC-MCMC algorithm proposed in [2] and refer to that paper for the algorithm. We are also inspired by the review in [3]. The algorithm requires a starting value θ_0, a proposal distribution, a distance measure between simulations and observations, and an acceptance threshold ϵ. We use starting values 1.2 for β_e and 5 for the assortativity. We assume an independent normal proposal distribution centred at the current value of the parameters, with variance 0.2 for β_e and 4 for the assortativity. We denote the proposal distribution by $P(x)$ where x is the current parameter value. As our distance measure, we compute the sum of the absolute deviance between the simulated and observed proportion of infected in ethnicity groups A and B. We use a threshold of $\epsilon = 0.001$. The idea is that we start with proposing $\theta_p \sim P(\theta_{i-1})$. If θ_p has 0 prior probability, we sample again. We then simulate with θ_p and compute the distance between the simulations and observations. If the distance is below ϵ, the parameters are accepted with a probability which depends on the proposal distribution and the prior, and we set $\theta_i = \theta_p$. Otherwise $\theta_i = \theta_{i-1}$. We use chains of length 1 000 000 and a burn-in of 100 000. As the true observations, we simulate from the model 100 times and hence perform 100 parameter calibrations in each setting.

S2. Supplementary results

S2.1 Infection dynamics in cases 1 and 2

S2.1.1 Case 1

The time series of infected for case 1 is provided in Figure S1, for some selected values of the reproduction number.

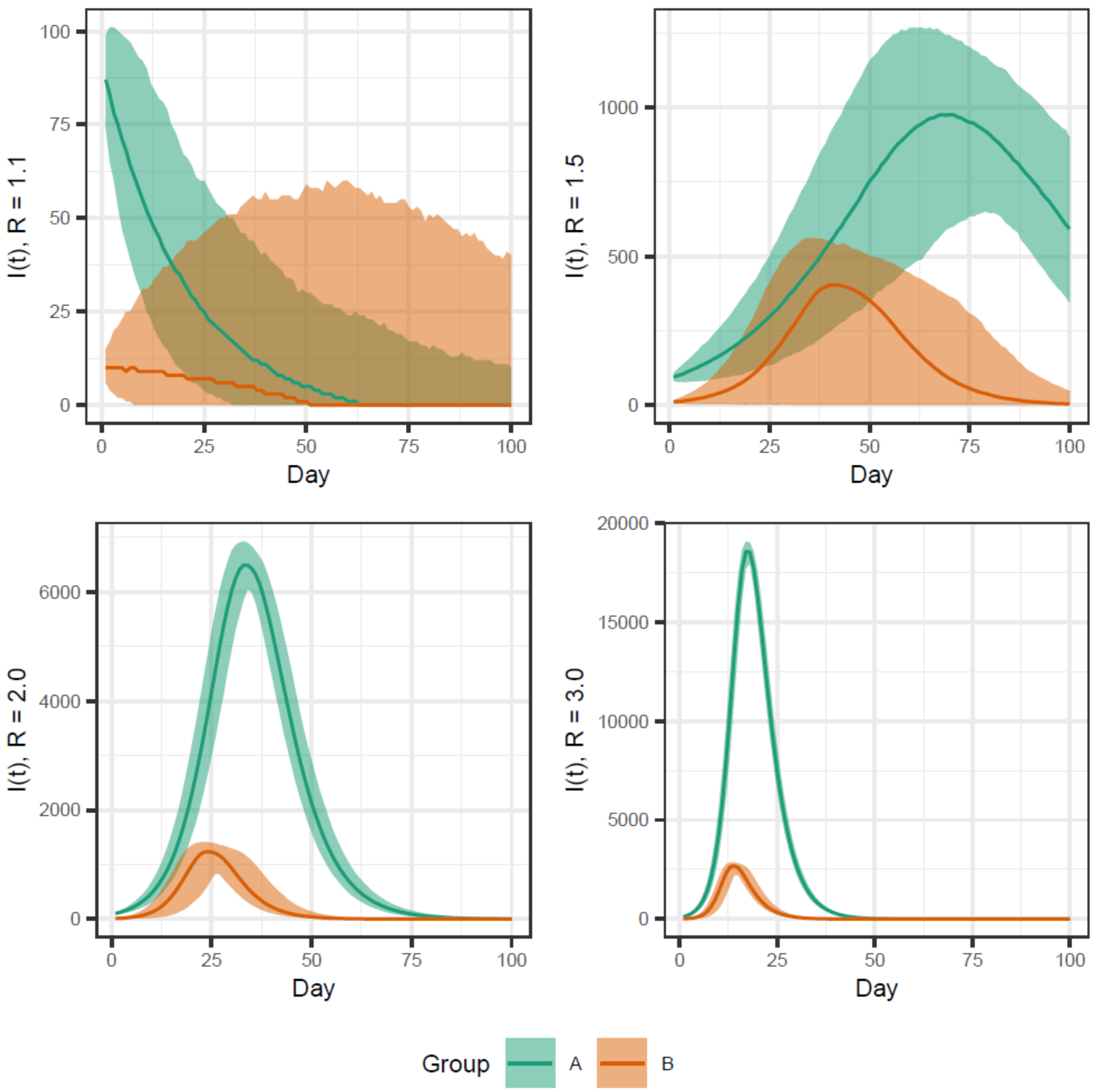

Figure S1. Time series of infected in groups A and B for $R_0 = 1.1, 1.5, 2.0$ and 3.0. The confidence intervals are based on 2000 simulations.

S2.1.2 Case 2

The time series of infected for case 2 is provided in Figure S2, for some selected values of the relative susceptibility in the high-risk group (A) to the low-risk group (B), a. We note that the disease dynamics and total number infected for the low-risk group depends on a, even though a only affects the individual-level risks in the high-risk group.

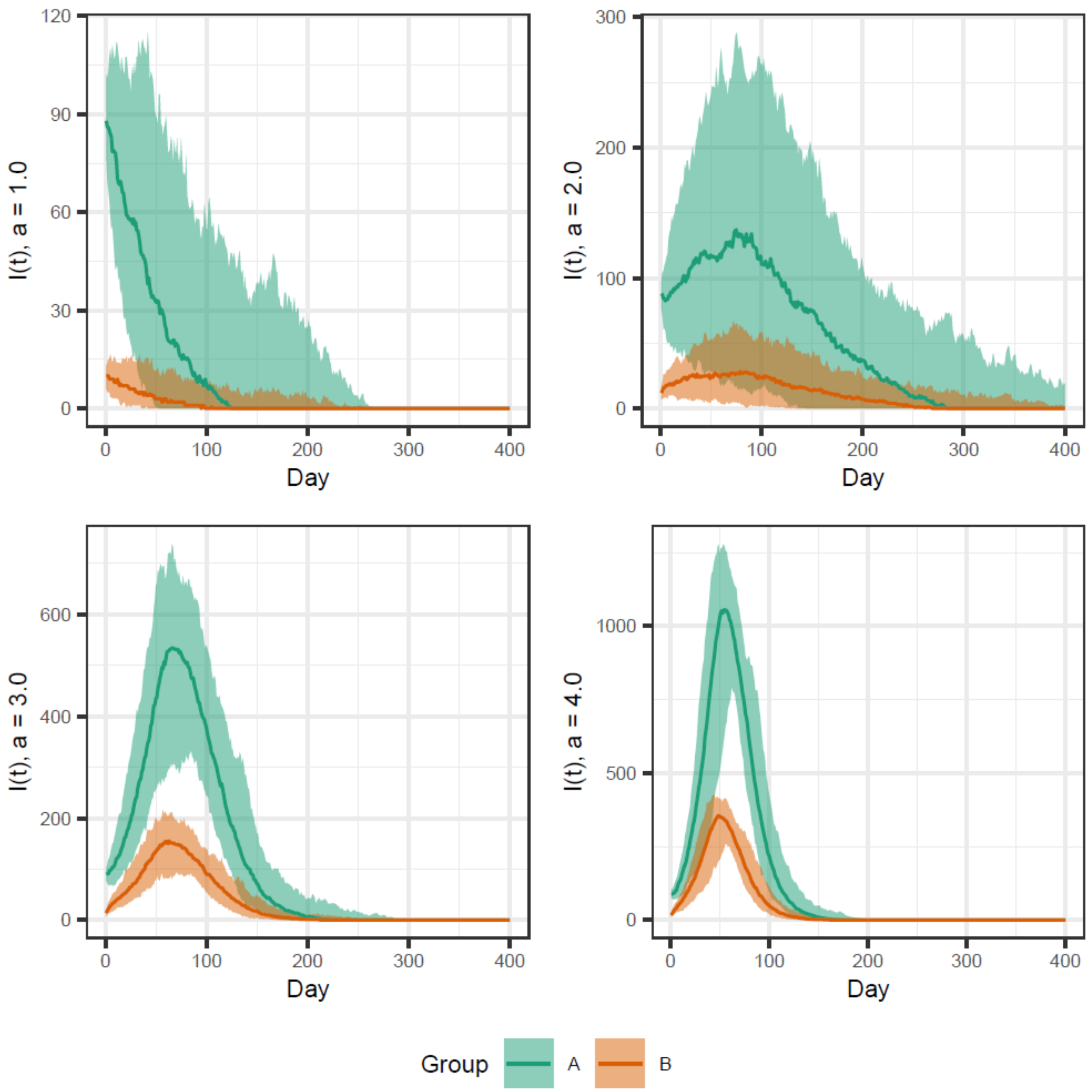

Figure S2. Time series of infected in groups A and B for $a = 1.0, 2.0, 3.0$ and 4.0. The confidence intervals are based on 2000 simulations.

S2.2 Results from ABC parameter estimation

We provide the results from the parameter estimation using ABC-MCMC. We study the setting where we estimate both the assortativity and β_e, and when we assume that the assortativity is known and estimate only β_e.

Figure S3 shows the histogram of estimated parameters in the different settings. The histogram is based on the 900 000 samples for each of the 100 simulations. We note that the estimated β_e is more accurate when we assume a known assortativity, but the mean value seems to be well captured in all four settings. We also note that the assortativity is not well estimated.

The estimated means together with 95% credible intervals for β_e in the four settings are 1.07 (1, 1.15), 1.05 (1, 1.1), 1 (0.95, 1.1), and 1 (0.96, 1.05) for the settings with true $\beta_e = 1.05$ and

assortativity unknown, $\beta_e = 1.05$ and known assortativity, $\beta_e = 1.0$ and assortativity unknown, and $\beta_e = 1.0$ and known assortativity, respectively. The mean and 95% credible interval for the assortativity when $\beta_e = 1.05$ is 8.9 (0.83, 19.2) and 8.8 (0.6, 19.3) when $\beta_e = 1.0$. We note that there is a strong, negative correlation between the estimated β_e and assortativity, in particular for $\beta_e = 1.05$. This is because both higher assortativity and higher β_e will result in more cases in ethnicity group 2 (when $\beta_e > 1$). The correlations are -0.71 for $\beta_e = 1.05$ and -0.55 for $\beta_e = 1.0$.

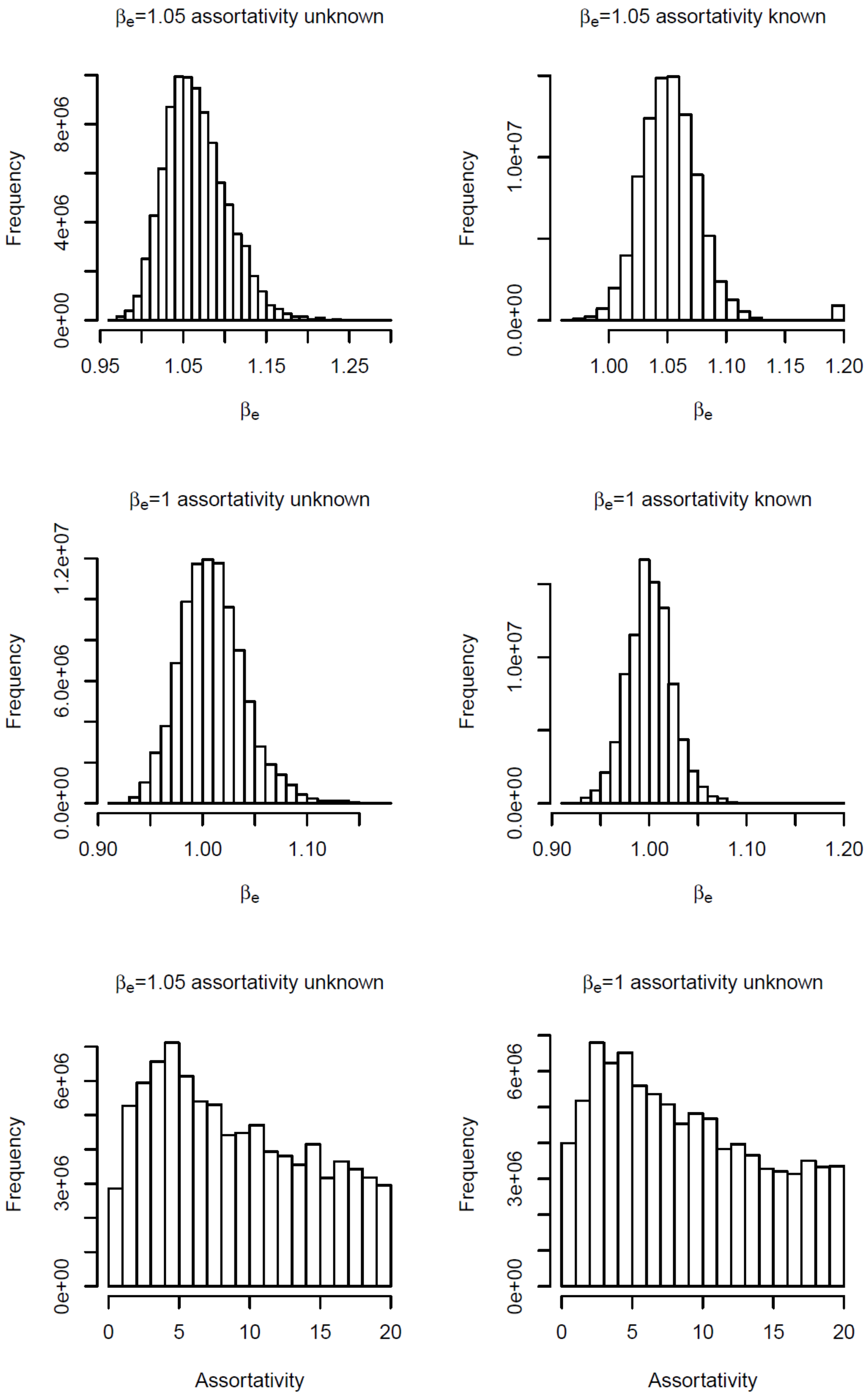

Figure S3. Estimated β_e for true values $\beta_e = 1.05$ and $\beta_e = 1$ and assortativity of 10, assuming both known and unknown assortativity. The lower panel shows the estimated assortativity.

The estimated β_e together with the 95%-credible interval from each simulation is provided in Figure S4. We note that most of the credible intervals are centred at the correct value, but also that the intervals vary between the simulations, most likely due to the stochasticity of the disease spread model.

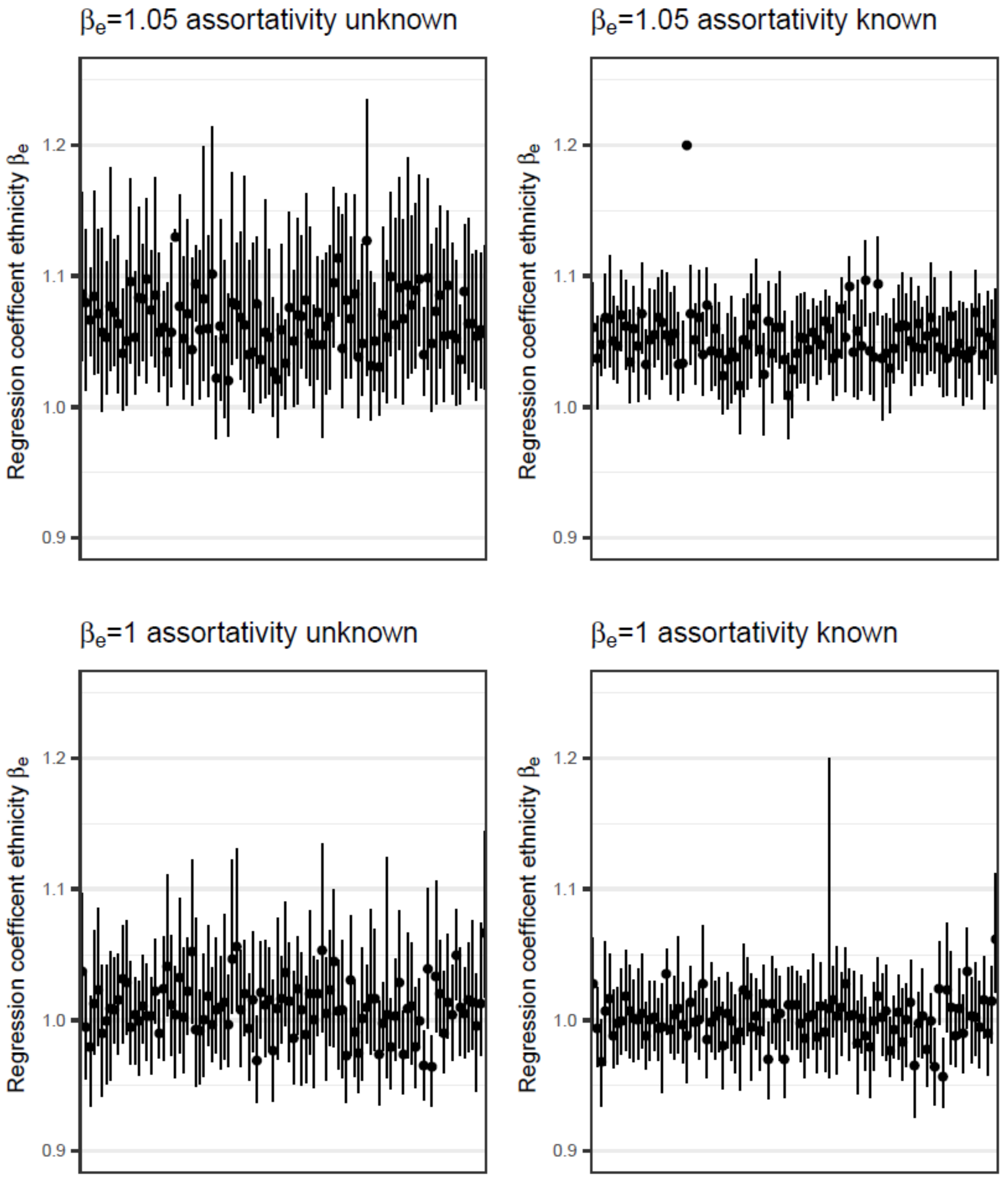

Figure S4. Estimated β_e and 95% credible interval for each of the 100 simulations.

References

1. Diekmann O, Heesterbeek JAP, Metz JA. On the definition and the computation of the basic reproduction ratio R 0 in models for infectious diseases in heterogeneous populations. *J Math Biol* 1990; 28(4): 365-382.

2. Marjoram P, Molitor J, Plagnol V et al. Markov chain Monte Carlo without likelihoods. *PNAS* 2003; 100(26): 15324-15328.
3. McKinley T, Cook AR, Deardon R. Inference in epidemic models without likelihoods. *Int J Biostat* 2009; 5(1): Article 24.
